# Multivariate Pattern Analysis of Lifelong Premature Ejaculation Based on Multiple Kernel Support Vector Machine

**DOI:** 10.3389/fpsyt.2022.906404

**Published:** 2022-07-25

**Authors:** Bowen Geng, Ming Gao, Ruiqing Piao, Chengxiang Liu, Ke Xu, Shuming Zhang, Xiao Zeng, Peng Liu, Yanzhu Wang

**Affiliations:** ^1^Life Science Research Center, School of Life Sciences and Technology, Xidian University, Xi’an, China; ^2^Engineering Research Center of Molecular and Neuro Imaging Ministry of Education, School of Life Sciences and Technology, Xidian University, Xi’an, China; ^3^Department of Urology, Xi’An Daxing Hospital Affiliated to Yan’an University, Xi’an, China

**Keywords:** machine learning, multivariate pattern analysis (MVPA), lifelong premature ejaculation (lifelong PE), neuroimaging, diffusion tensor imaging (DTI), MRI, support vector machine (SVM)

## Abstract

**Objective:**

This study aimed to develop an effective support vector machine (SVM) classifier based on the multi-modal data for detecting the main brain networks involved in group separation of premature ejaculation (PE).

**Methods:**

A total of fifty-two patients with lifelong PE and 36 matched healthy controls were enrolled in this study. Structural MRI data, functional MRI data, and diffusion tensor imaging (DTI) data were used to process SPM12, DPABI4.5, and PANDA, respectively. A total of 12,735 features were reduced by the Mann–Whitney U test. The resilience nets method was further used to select features.

**Results:**

Finally, 36 features (3 structural MRI, 7 functional MRI, and 26 DTI) were chosen in the training dataset. We got the best SVM model with an accuracy of 97.5% and an area under the curve (AUC) of 0.986 in the training dataset as well as an accuracy of 91.4% and an AUC of 0.966 in the testing dataset.

**Conclusion:**

Our findings showed that the majority of the brain abnormalities for the classification was located within or across several networks. This study may contribute to the neural mechanisms of PE and provide new insights into the pathophysiology of patients with lifelong PE.

## Introduction

Premature ejaculation (PE) is one of the most common male sexual dysfunction worldwide. According to the International Society for Sexual Medicine and European Association of Urology Guidelines, the definition of lifelong PE was characterized by the following: ejaculation that always or nearly always occurs prior to or within about 1 min of vaginal penetration (1). Consistent with epidemiological data, there is approximately a 5% lifetime prevalence of PE in the general population (1). PE may involve multiple etiologies, including genetics, neurobiology, endocrinology, urology, psychology, and related factors (2). In recent years, the neural mechanism of PE has gradually attracted people’s attention. Undoubtedly, it is of great significance to investigate the objective neurobiological markers for diagnosis and treatment.

The development of neuroimaging techniques, such as structural and functional MRI (fMRI) as well as diffusion tensor imaging (DTI), provides a task-free approach and a reliable measure of the brain mechanism. A significant difference in the caudate nucleus volume was found between patients with PE and healthy controls (HCs) (3). Patients with lifelong PE also had altered gray matter volume (GMV) in the bilateral amygdala (4). A recent study found that altered amplitude of low-frequency fluctuations (ALFF) of the frontal cortex, the parietal cortex, and the putamen might help distinguish premature ejaculation from anejaculation (5). Another study investigated the neural basis of patients with PE based on the measure of regional homogeneity (ReHo) (6). Patients with lifelong PE showed abnormal degree centrality (DC) value in the medial prefrontal cortex, the precuneus, the primary somatosensory cortex, and the orbitofrontal cortex (7). Compared to HCs, patients with PE also showed widespread increases in fractional anisotropy (FA) and axial diffusivity values (8). Although these studies undoubtedly provided meaningful insights into brain functional and structural abnormalities of PE underlying traditional intergroup statistical methods, most of these studies applied single or two types of neuroimaging data and involve a few indicators for comparison, and the method that can effectively integrate multiple types of brain images and multiple brain indicators are still unclear.

Multivariable pattern analysis (MVPA) based on machine learning could better utilize the inherent multivariable properties of high-dimensional neuroimaging data to classify new samples, which works by decoding patterns of differences between brain regions/connections by learning discriminant rules from datasets (9). As one of the many MVPA methods, SVM is one of the most prevalent classifiers used in neuroimaging-based classification, and SVM models can provide confidence in the classification in terms of the distance to the separating hyperplane (10). A previous study indicated that the integration of structural MRI (sMRI), fMRI, and DTI may provide more energy for the diagnosis of schizophrenia (11). Other findings suggest that machine learning classifiers trained based on resting-state fMRI features of participants under anesthesia may help to distinguish the degree of pathological unconsciousness in clinical patients (12). Although a previous studiy applied an SVM model in fMRI data to distinguish patients with PE from HCs (13), there have been no reports combining three types of brain imaging data including several structural and functional measures to investigate the brain mechanism of PE, and the pathophysiology and neural mechanisms of abnormal brain regions in patients with PE need further study.

Against this background, we aimed to (1) develop an effective SVM classifier based on the three types of neuroimaging data and (2) detect the main brain networks involved in group separation in order to improve the understanding of PE.

## Materials and Methods

### Participants

A total of 88 right-handed adult men including 52 drug-naïve patients with lifelong PE and 36 matched HCs were enrolled in the present study. Patients with lifelong PE were diagnosed by the guideline of the International Society for Sexual Medicine (ISSM) (1). For the inclusion criteria of all patients with lifelong PE, the Premature Ejaculation Diagnostic Tool (PEDT) score was >11, the intravaginal ejaculation latency time (IELT) was <1 min, and the International Index of Erectile Function (IIEF-5) score was >21 (14). The inclusion criteria of HCs were as follows: (1) PEDT was <5; (2) IELT > 3 min; and (3) IIEF-5 score > 21. All participants with a history of neurological or psychiatric disorders, of urological surgery, and of alcohol, nicotine, or drug abuse were excluded from this study. Self-Rating Anxiety Scale (SAS) and Self-Rating Depression Scale (SDS) were utilized to examine the anxiety and depression \52\ 28.

### MRI Data Acquisition

Individual MRI data were acquired using a 3T MRI system (Excite, General Electric, Milwaukee, WI, United States). Participants were asked to lie in the scanner in the supine position, with their eyes closed and without thinking, and the head motion were restricted with foam fillers. The functional MRI images were collected by using the gradient echo with the following parameters: repetition time (TR) = 2,000 msec, echo time (TE) = 30 ms, slice thickness = 3.5 mm, data matrix = 64 × 64, flip angle (FA) = 90°, field of view (FOV) = 240 mm × 240 mm, in-plane resolution = 3.75 mm × 3.75 mm. The scan parameters of the T1 images were as follows: TR = 8.2 ms; TE = 3.2 ms; FA = 12°; data matrix = 256 × 256; in-plane resolution = 1 mm × 1 mm; FOV read = 256 mm; and slice thickness = 1 mm. The scan parameters of DTI data were as follows: TR/TE = 10,000/85.3 ms, FOV = 240 mm × 240 mm, slice thickness = 2 mm, data matrix = 256 × 256, and 70 continuous axial slices with no gap. 2 Diffusion-weighted sequences were acquired using gradient values *b* = 0 and *b* = 1,000 s/mm^2^ with diffusion sensitizing gradients applied along 64 non-linear directions.

### Structural MRI Preprocessing

The T1-weighted structural data analysis was preprocessed by the CAT12 toolbox, which is based on Statistical Parametric Mapping 12 (SPM12)^1^ in accordance with the following procedures: (1) T1 data was normalized to the template space; (2) the processed images were segmented into gray matter (GM), white matter (WM), and cerebrospinal fluid (CSF); (3) quality checks, including displaying one slice for all images and checking sample homogeneity, were carried out; (4) different brain sizes based on total intracranial volume (TIV) by the ANCOVA model were corrected; and (5) the image data with an 8 mm full-width at half-maximum Gaussian kernel were smoothed (4).

### Functional MRI Preprocessing

The preprocessing of the fMRI imaging data was accomplished by the DPABI toolbox (DPABI 4.5).^2^ First, the first five volumes to avoid the effect of the scanning inadaptation were removed. Then, slice-timing and head motion were corrected for the time delay of acquisition and the alignment to the first volume, respectively. We excluded all subjects whose maximum translation was larger than 2 mm or head rotation exceeded 2°. After normalizing to the Montreal Neurological Institute (MNI) space by using the EPI template, images were resampled at 3 mm × 3 mm × 3 mm. The nuisance factors included 24 head motion parameters, the WM, and the CSF signal in the procedure of nuisance covariates regression. Finally, smoothing, detrending, and temporal bandpass filtering (0.01–0.1 Hz).

### Diffusion Tensor Imaging Preprocessing

Diffusion tensor imaging (DTI) data were processed using the PANDA, which was a pipeline toolbox for analyzing brain diffusion imaging. The individual FA images of native space were registered to the FA template in the MNI space. After that, the resulting warping transformation was applied to write the diffusion metric image, such as mean diffusivity (MD), axial diffusivity (AD), and radial diffusivity (RD), into MNI space. In this study, the Anatomical Automatic Labeling (AAL) template with 90 cortical and subcortical regions (45 in each hemisphere) was applied to obtain the mean tensor-based parameters value of each brain region. For network construction, the whole-brain fiber tractography was obtained by a deterministic streamline tracking algorithm. If it turns at an angle of more than 45° or reaches a voxel with a FA < 0.15, the tractography terminates. In addition, the FA matrix, the fiber number (FN) matrix, and the fiber length matrix were computed for network analysis.

### Feature Extraction and Feature Selection

In the current study, we calculated functional and structural measures, including GMV, ReHo, ALFF, DC, FA, MD, AD, and RD, as well as a matrix of the mean value of FA on the fiber, a matrix of the fiber length, and a matrix of the fiber number as the variables in the following statistical analysis. First, we used a *z*-score to standardize all the features. Then, a two-stage feature selection technique was used to select the most discriminating features and improve the reliability of the results. Since the number of matrix features (FA matrix, fiber number, fiber length) is much larger than other features, a non-parametric Mann–Whitney U test was used for preliminary selection to make all feature dimensions similar (40 features with minimum *p*-value were retained for each type). Considering individual variation, in order to ensure the stability of the retained variables, we adopted the keep-one elimination method. After the Mann–Whitney U test in the first step, we spliced the remaining features with other features as the input for the second feature selection. When applying L1-penalty and L2-penalty simultaneously, the elastic net exhibited the grouping effect, i.e., selecting a group of features with a high correlation and making their regression coefficients to be equal. Due to the simultaneous application of L1- and L2-penalties, the elasticity network exhibits a grouping effect, which is a set of features with high correlation selected so that their regression coefficients tend to be equal. Therefore, 4-fold cross-validation resilience nets were randomly shuffled 100 times with the classification accuracy as a cost function to select the most informative set of relevant features and retain non-zero features.

### Support Vector Machine Classifier

**FIGURE 1:**
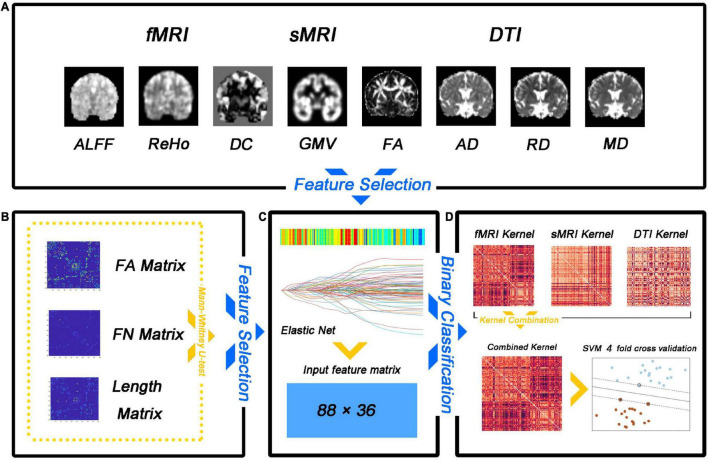
Flow diagram of the analysis approach employed in the study. **(A)** Feature selection. **(B)** The number of matrix features (FA matrix, fiber number, and fiber length) was reduced by the non-parametric Mann–Whitney U test. **(C)** The feature matrix is extracted after dimensionality reduction. **(D)** The multi-kernel support vector machine (SVM) model training.

A fourfold cross-validation strategy was used to evaluate the classification performance, which included accuracy, sensitivity, specificity, precision, recall, and f1. Specifically, all samples were divided equally into four subsets, with samples from three subsets being used to train a multi-kernel SVM classifier and samples from another subset being used to calculate classification metrics. The entire dataset was randomly shuffled 100 times to avoid any bias introduced by randomly splitting the dataset, and cross-validation was performed after each shuffle. Average classification accuracy, sensitivity, specificity, precision, recall f1, and area under the curve (AUC) were calculated over the 100 runs. The flow diagram for this study is illustrated in Figure 1. Our method was implemented using the Scikit-learn and Scipy packages for Python. Overall, the performance of our method is assessed by various metrics including accuracy, sensitivity, specificity, precision, recall, f1, the receiver operating characteristic (ROC) curve, and AUC.

## Results

### Demographics and Clinical Results

The demographics and clinical features (age, PEDT, IELT, SAS, and SDS) of all participants are summarized in Table 1.

**TABLE 1:** Demographic and clinical features of patients with lifelong PE and HCs.

	HC (*n* = 36)	PE (*n* = 52)	*P*-value
Ages (years)	31.69 ± 0.47	30.40 ± 0.75	0.1923
IIEF-5 score	24.17 ± 0.22	23.83 ± 0.12	0.1426
IELT (min)	10.72 ± 0.87	0.6500 ± 0.03	0.000***
PEDT	1.028 ± 0.32	16.98 ± 0.25	0.000***
Anxiety	30.50 ± 0.27	39.08 ± 0.82	0.000***
Depression	30.67 ± 0.36	40.81 ± 0.46	0.000***

*PE, premature ejaculation; HC, healthy controls; IIEF-5, International Index of Erectile Function; IELT, intravaginal ejaculatory latency time; PEDT, premature ejaculation diagnostic tool.*

***, P < 0.001 by a two-sample t-test.

### Feature Selection

**FIGURE 2:**
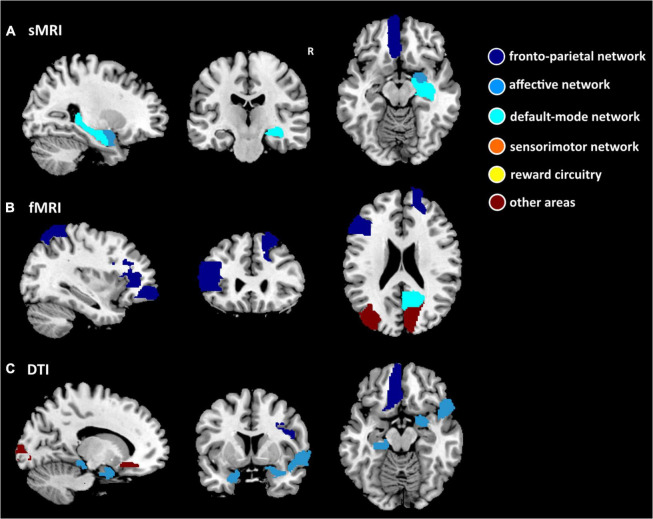
Nineteen features separately of structural MRI (sMRI) (GMV), fMRI (ReHo, ALFF, DC, and FC), and DTI (FA, MD, AD, and RD) data for discriminating between patients with PE and healthy control subjects. **(A)** Three ROIs have the smallest *p*-value for the sMRI measure. **(B)** Seven ROIs have the smallest *p*-value for fMRI measures. **(C)** Nine ROIs have the smallest *p*-value for the DTI measure. The dark blue region indicates the frontoparietal network, the light blue region indicates the affective network, the indigo region indicates the default-mode network, the orange region indicates the sensorimotor network, the yellow region indicates the reward circuitry, and the dark red region indicates the other areas in the AAL template.

**FIGURE 3:**
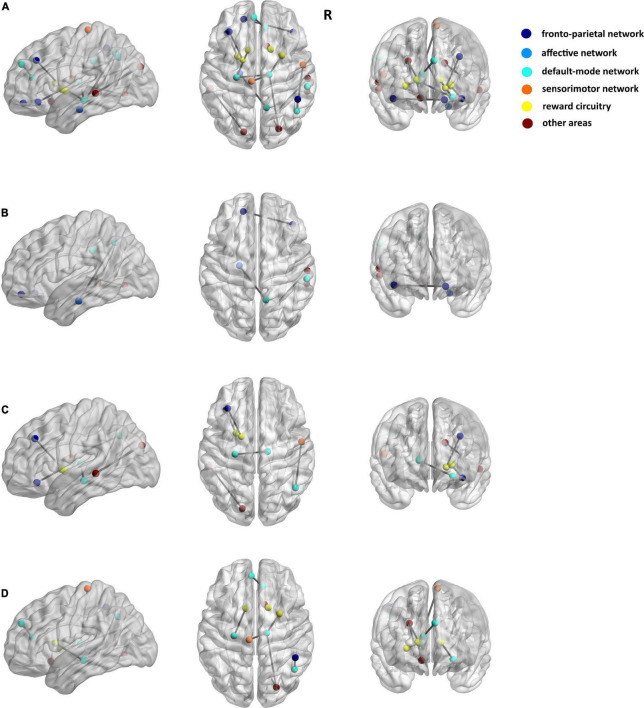
Seventeen matrix features of DTI data for discriminating patients with PE and healthy control subjects. **(A)** The results of the FA matrix, the FN matrix, and the length matrix. **(B)** Only FA matrix. **(C)** Only FN matrix. **(D)** Only Length matrix. The dark blue spot indicates the frontoparietal network, the light blue spot indicates the affective network, the indigo spot indicates the default-mode network, the orange spot indicates the sensorimotor network, the yellow spot indicates the reward circuitry, and the dark red spot indicates the other areas in the AAL template.

Among the 3 types of data, 12,735 features were reduced by the Mann–Whitney U test. Finally, 36 features (3 sMRI, 7 fMRI, and 26 DTI) were chosen in the training dataset. Supplementary Table 1 shows the 36 features in detail with the AAL template. These abnormalities were located within or across the default mode network, the affective network, the sensorimotor network, the reward circuitry, and the frontoparietal network (Figures 2, 3).

### Support Vector Machine Analysis

**FIGURE 4:**
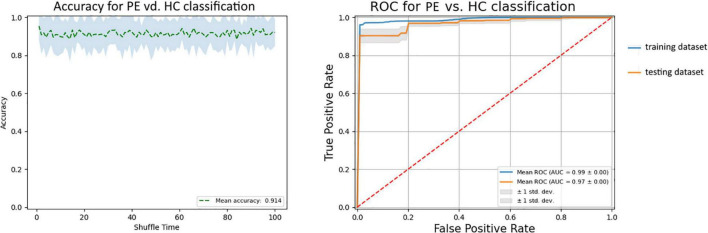
The ROC curve of the SVM model. The blue line is the ROC curve of the training dataset, and the orange line is the ROC curve of the testing dataset. ROC, receiver operating characteristic.

After training SVM in the training dataset with the 36 selected features, we got the best SVM model with an accuracy of 97.5% and an AUC of 0.986 in the training dataset. Then, we used the model to predict the status in the testing dataset with an accuracy of 91.4% and an AUC of 0.966 (Figure 4). The results showed that 36 selected features could distinguish the difference between patients with PE and the general population.

## Discussion

Within this study, we applied a multivariate pattern analysis to distinguish patients with lifelong PE from HCs. The accuracy was 97.5% and the AUC was 0.99 in the training dataset by applying 36 selected features in 3 types of data, and the accuracy was 91.4% and the AUC was 0.97 in the testing dataset. To the best of our knowledge, this is the first study to develop a multivariate pattern method that combines the resting state sMRI, fMRI, and DTI data in the classification of PE and HCs. Our results showed that the majority of the structural and functional abnormalities that contributed to the classification were located within or across the default mode network, the affective network, the sensorimotor network, the reward circuitry, and the frontoparietal network.

Previous studies reported their works on functional or structural abnormalities by using traditional group-level statistical methods. A notable finding using the SVM model has shown important progress in distinguishing patients with PE from HCs (13). The researchers found significant group differences in functional connectivity (FC) in patients with PE between the (1) left and right orbitofrontal cortices, (2) the left rectus and right postcentral gyri, (3) the right insula and the left pallidum, and (4) the right middle part of the temporal pole and the right inferior part of the temporal gyrus. Although the classification model showed good performance (accuracy = 0.85 ± 0.14, AUC = 0.8), their study only involved single-modal resting-state fMRI data (13). By contrast, our proposed method applied machine learning methods to investigate the neural mechanism of PE by combining three types of brain imaging data with several structural and functional measures.

The feature selection process discerned the functional and structural abnormalities in PE largely covering the default mode network and the sensorimotor network. The functional elements of the default mode network comprise supporting emotional processing, self-referential mental activity, and the recollection of prior experiences (15). The relevant brain regions involved in the current study included the precuneus (16), the hippocampus, the thalamus, the medial part of the superior frontal gyrus, and the angular gyrus, which have been previously reported to be associated with PE (17). For instance, a previous study found a significantly decreased short-range functional connectivity density in the thalamus and increased long-range functional connectivity density in the precuneus (18). Patients with PE also had decreased FC between the left nucleus accumbens and the bilateral thalamus (19). A DTI study using nodal strength investigated the difference in group-specific hub regions, including the precuneus and hippocampus, as well as increased global efficiency, strength, and decreased shortest path length in the medial part of the superior frontal gyrus (8). In addition, our results also suggest that the sensorimotor network may have a role in distinguishing patients with PE from HCs. Several regions are known to be reported in the recent study (20). A recent neuroimaging study showed that abnormal neural activation responses to visual erotic stimulation in patients with lifelong PE were related to the sensorimotor processing region (21). Patients with lifelong PE had increased DC value in the primary somatosensory cortex (7). Taken together, these findings suggest that an altered default mode network and the sensorimotor network may provide a reliable neuroimaging-based interpretation of PE, which may indicate that the abnormalities in the default mode network and the sensorimotor network are related to defective function of self-referential processing, self-focused attention, and sensory information processing in patients with PE.

The observed group differences in the reward circuit and the affective network are particularly noteworthy when comparing patients with PE with HCs. The reward circuit comprises the ventral tegmental area dopaminergic neurons, which innervate several regions of the part of the ventral striatum, the prefrontal cortex, the amygdala, and the hippocampus, as well as other areas (22). It plays an important role in the recognition of environmental rewards, initiation of consumption, and response to aversive stimuli (23). Previous studies reported these so-called brain reward regions in patients with PE compared to HCs (3, 24). Especially, structural covariance changes of the striatum were found in patients with lifelong PE (25). Patients with PE also had significantly increased mean caudate nucleus volume, while the decreased mean amygdala volume was shown in patients compared to HCs (3, 4). In other respects, previous studies reported structural and functional abnormalities of the abovementioned regions in patients with PE. As expected, the current results involve several regions in the affective network, such as the amygdala, the temporal pole, the pallidum, and the parahippocampal cortex. In particular, the amygdala is considered the basis for emotion regulation processes (26) and also is connected in complex ways with other reward regions (22). Further, in our results, the features of the amygdala both in sMRI (GMV) and DTI (RD) were available as biomarkers for PE. We thereby speculated that the pathophysiology of PE might be related to the ineffective emotional regulation process and integration of reward information. In this case, these findings may provide us with a better understanding of the neural mechanism of PE.

Our main analysis of features revealed that certain regions located in the frontoparietal network (including the prefrontal cortex and lateral parietal cortices) and other regions (including temporal and occipital cortex) were associated with PE. The control system, consisting of several sub-systems (“frontoparietal,” “cingulo-opercular,” and “dorsal attention” portion), is considered to be involved in highly adaptive control processes, time-extended control processes, and coordinated attention to external stimuli (27). It is widely believed that the control system, especially the frontoparietal network, can communicate extensively with a variety of systems throughout the brain (28). A prevalent view is that the prefrontal cortex serves as a source of inhibitory control over other brain areas (29). According to previous studies about PE, multiple brain regions with abnormalities of function and structure were in the frontoparietal network. For example, patients with lifelong PE had decreased amygdala-related FC in the inferior frontal gyrus, which is a specialized response inhibition area (4). The increased DC value in the medial prefrontal cortex was found in patients with PE compared with HCs (7). Patients with PE also had decreased FC between the nucleus accumbens and the orbitofrontal cortex (19). In our study, the abnormalities in the frontoparietal network might reflect the information that patients with PE had abnormal processing of extending the control process, coordinating attention to external stimulation, and inhibitory controls during ejaculation. Further, there are also other regions worthy of attention reported in our study. The findings suggested that the temporal and occipital cortices were associated with PE, which was mentioned by previous studies (30).

The current study has several limitations. First, although the sample of our study was larger than previous studies, the sample size was relatively small. Second, future studies should use longitudinal data that may reduce possible confounders. Finally, besides the AAL-90 atlas, future studies should further detect subtle anatomical differences between different atlases.

## Conclusion

In conclusion, based on three types of imaging data and machine learning approaches, our study showed that the majority of the brain abnormalities for the classification were located within or across several networks. This study may contribute to neural mechanisms associated with PE and provide new insights into the pathophysiology of patients with lifelong PE.

## Data Availability Statement

The original contributions presented in this study are included in the article/Supplementary Material, further inquiries can be directed to the corresponding authors.

## Ethics Statement

The studies involving human participants were reviewed and approved by the Northwest Women’s and Children’s Hospital Ethics Committee. The patients/participants provided their written informed consent to participate in this study. Written informed consent was obtained from the individual(s) for the publication of any potentially identifiable images or data included in this article.

## Author Contributions

BG and PL: conceptualization. MG and YW: data curation. BG and RP: methodology. MG, CL, SZ, and KX: investigation. RP, CL, and XZ: software. BG: writing – original draft. BG, YW, PL, and RP: writing – review and editing. MG: funding acquisition. YW, PL, and XZ: resources. All authors contributed to the article and approved the submitted version.

## Conflict of Interest

The authors declare that the research was conducted in the absence of any commercial or financial relationships that could be construed as a potential conflict of interest.

## Publisher’s Note

All claims expressed in this article are solely those of the authors and do not necessarily represent those of their affiliated organizations, or those of the publisher, the editors and the reviewers. Any product that may be evaluated in this article, or claim that may be made by its manufacturer, is not guaranteed or endorsed by the publisher.

## Footnotes

1
http://www.fil.ion.ucl.ac.uk/spm

2
http://rfmri.org/dpabi
