# Supplementary material for: Multivariate Pattern Analysis of Lifelong Premature Ejaculation Based on Multiple Kernel Support Vector Machine
Source: Front Psychiatry. 2022 Jul 25;13:906404. doi: 10.3389/fpsyt.2022.906404 (PMC9357875; doi:10.3389/fpsyt.2022.906404)
Supplement: Supplementary file 1 [file Table_1.DOCX]

Supplementary Material

**Supplementary Table 1.** The features for discriminating the lifelong PE group and HC group.

| **ID** | **Brain region** | **AAL number** | **Network** |  | **ID** | **Brain region A** | **AAL number** | **Network** | **Brain region B** | **AAL number** | **Network** |
| --- | --- | --- | --- | --- | --- | --- | --- | --- | --- | --- | --- |
| **ALFF** | | | |  | **FA matrix** | | | | | | |
| **1** | Frontal_Inf_Tri_L | 13 | FPN |  |  |  |  |  |  |  |  |
| **2** | Cuneus_R | 46 | Other region |  | **20** | Frontal_Inf_Orb_R | 16 | FPN | Frontal_Sup_Orb_L | 5 | FPN |
| **3** | Occipital_Mid_L | 51 | Other region |  | **21** | Precuneus_R | 68 | DMN | ParaHippocampal_L | 39 | AN |
| **4** | Precuneus_R | 68 | DMN |  | **22** | Temporal_Sup_R | 82 | Other | Lingual_R | 48 | Other |
| **ReHo** | | | |  | **23** | Temporal_Mid_R | 86 | Other | SupraMarginal_R | 64 | DMN |
| **5** | Frontal_Mid_Orb_L | 9 | FPN |  |  |  |  |  |  |  |  |
| **6** | Parietal_Sup_L | 59 | FPN |  |  |  |  |  |  |  |  |
| **DC** | | | |  | **fiber number matrix** | | | | | | |
| **7** | Frontal_Sup_R | 4 | FPN |  |  |  |  |  |  |  |  |
| **FA** | | | |  | **24** | Angular_R | 66 | DMN | Rolandic_Oper_R | 18 | SMN |
| **8** | ParaHippocampal_L | 39 | AN |  | **25** | Putamen_L | 73 | RC | Frontal_Inf_Orb_L | 15 | FPN |
| **9** | Occipital_Mid_L | 51 | Other region |  | **26** | Pallidum_L | 75 | RC | Frontal_Mid_L | 7 | FPN |
| **AD** | | | |  | **27** | Thalamus_R | 78 | DMN | Hippocampus_L | 37 | DMN RC |
| **10** | Temporal_Pole_Sup_R | 84 | AN |  | **28** | Temporal_Mid_L | 85 | Other | Occipital_Sup_L | 49 | Other |
| **MD** | | | |  |  |  |  |  |  |  |  |
| **11** | Frontal_Inf_Oper_R | 12 | FPN |  |  |  |  |  |  |  |  |
| **12** | Temporal_Pole_Sup_R | 84 | AN |  | **length matrix** | | | | | | |
| **RD** | | | |  |  |  |  |  |  |  |  |
| **13** | Frontal_Inf_Oper_R | 12 | FPN |  | **29** | Cingulum_Ant_R | 32 | DMN | Frontal_Sup_Medial_L | 23 | DMN |
| **14** | Rectus_L | 27 | Other region |  | **30** | Occipital_Sup_R | 50 | Other | Lingual_R | 48 | Other |
| **15** | Amygdala_R | 42 | AN,RC |  | **31** | Angular_R | 66 | DMN | Parietal_Inf_R | 62 | FPN |
| **16** | Temporal_Pole_Sup_R | 84 | AN |  | **32** | Caudate_L | 71 | RC | Hippocampus_L | 37 | DMN,RC |
| **GMV** | | | |  | **33** | Caudate_R | 72 | RC | Olfactory_R | 22 | Other |
| **17** | Frontal_Med_Orb_L | 25 | FPN |  | **34** | Thalamus_R | 78 | DMN | Occipital_Sup_R | 50 | Other |
| **18** | Hippocampus_R | 38 | DMN,RC |  | **35** | Thalamus_R | 78 | DMN | Paracentral_Lobule_L | 69 | SMN |
| **19** | Amygdala_R | 42 | AN,RC |  | **36** | Thalamus_R | 78 | DMN | Putamen_R | 74 | RC |

**Abbreviations:** PE, premature ejaculation; HC, healthy controls; ReHo, regional homogeneity; ALFF, amplitude of low-frequency fluctuations; DC ,degree centrality; FC, functional connectivity; GMV, gray matter volume; FA, fractional anisotropy; MD, mean diffusivity; AD, axial diffusivity ; RD, radial diffusivity. DMN, default mode network; AN, affective network; SMN, sensorimotor network; RC, reward circuitry; FPN, fronto-parietal network.
